# Psychosocial risks and mental health of preschool care providers in Kuala Lumpur, Malaysia: a cross-sectional study

**DOI:** 10.1186/s40359-024-02279-3

**Published:** 2025-01-09

**Authors:** Sin Wan Tham, Min Fui Wong, Maslinor Binti Ismail, Noriklil Bukhary Binti Ismail Bukhary

**Affiliations:** 1 Centre for Burden of Disease Research, Institute for Public Health, National Institutes of Health, Blok B2, Kompleks Institut Kesihatan Negara (NIH), No.1, Jalan Setia Murni U13/52, Seksyen U13 Setia Alam, Shah Alam, Selangor 40170 Malaysia; 2 Centre for Health Policy Research, Institute of Health System Research, National Institute of Health, Kompleks Institut Kesihatan Negara (NIH), No.1, Jalan Setia Murni U13/52, Blok B2, Seksyen U13 Setia Alam, Shah Alam, Selangor 40170 Malaysia; 3 Department of Social and Preventive Medicine, Faculty of Medicine, University Malaya, Wilayah Persekutuan Kuala Lumpur, Kuala Lumpur, 50603 Malaysia; 4 Health Department of Kuala Lumpur and Putrajaya, Health office of Lembah Pantai District, Ministry of Health, Kuala Lumpur, Malaysia

**Keywords:** Mental health, Psychosocial, Job strain, Preschool care-providers

## Abstract

**Background:**

Child maltreatment in daycare is a public health issue. As childcare is stressful, high care provider negativity independently predicts more internalizing behaviour problems, affecting children’s psycho-neurological development. This study aimed to determine psychosocial factors associated with the mental health of preschool care providers in Kuala Lumpur.

**Methods:**

A random cluster sampling cross-sectional survey was conducted in 2019. The study involved registered care providers for preschoolers under four years old without acute psychiatric illness. The Center for Epidemiologic Studies Depression Scale (Malay-CES-D) and Karasek’s Job Content Questionnaires (Malay-JCQ) were used to assess depression symptoms and psychosocial job-related risks. Logistic regression (*p* < 0.05) guided by the Hosmer-Lemeshow strategy was used for analysis, with the final model evaluated for assumptions and goodness-of-fit.

**Results:**

A total of 247 providers were recruited from 36 preschools. All respondents were females, with a mean age of 32.1 years old, Malays (70.4%), married (55.0%), attained a diploma and above (50.6%) and had low income (80.1%). The prevalence of depressive symptoms and job strain was 28.7%. Final logistic regression revealed individual factors (married, stressful life events: assault and marital issues), job strain (AOR = 2.33, CI = 1.22, 4.44), and job insecurity (AOR = 1.29, CI = 1.07, 1.56) determine depressive symptoms. Good supervisor support was inversely associated with depression.

**Conclusions:**

Job strain and insecurity contribute significantly to depressive symptoms among preschool care providers in Kuala Lumpur. The Social and Welfare Department can promote supportive supervision through skill training for supervisors, fostering a positive mental health environment for improving workers’ mental health.

**Supplementary Information:**

The online version contains supplementary material available at 10.1186/s40359-024-02279-3.

## Introduction

Family structures and dynamics have undergone remarkable paradigmatic shifts over the years [1–3]. Throughout the 21st century, a transformation in family structures has been observed, featuring an increase in dual-income households and single-parent families [4–6]. The growing number of working parents led to a significant rise in demand for childcare services. Furthermore, the rising availability of education and employment opportunities is leading to a higher proportion of women joining the workforce [7, 8], which increases the demand for childcare centres worldwide, including Malaysia [9, 10].

In this era, childcare services are an indispensable requirement for families [11, 12]. Thus, ensuring the safety and development of children is paramount. However, the alarming number of child daycare maltreatment cases poses a risk, with some incidents resulting in fatal outcomes for the children. There is growing evidence of its occurrence not only on a global scale [13–15] but also in Malaysia [12, 16]. In 2022, the Social and Welfare Department reported a rise in child abuse cases, increasing from 3,831 in 2012 to 6,770 [17]. This issue has been recognized as a major global public health concern [18, 19], as it directly impacts the safety of vulnerable children [20–22] and hindrances to their psychosocial development, resulting in enduring physical and psychological consequences [14, 23, 24]. Additionally, it gives rise to broader societal and economic implications [25–27].

Indeed, good mental health among childcare providers is vital to ensure good quality care for the children. Hamre and Pianta (2004) revealed that depressed childcare workers exhibit lower sensitivity and increased withdrawal compared to their non-depressed counterparts [28]. The burden of mental health issues among these providers is substantial, ranging from 6 to 47.5% [29, 30]. The role of a childcare provider is challenging, with job strain strongly linked to the development of depression in working adults [31–34]. A recent longitudinal study revealed that high job demands coupled with low social support over the long term increase the risk of depression [35]. Rafferty et al. (2001) conducted a study to assess the influence of psychosocial risk factors on burnout. The findings suggested that social support does not moderate the effects of high job demand, job control, and burnout [36]. Teachers with higher levels of training were found to moderate teacher depression by reducing harshness and a tune towards children’s behaviour [37]. Another longitudinal study done in Danish using SF-36 to screen for depression found that perceived job insecurity among women is associated with an increased risk of depression [38]. Traumatic life events and chronic illness are established predictors of mental illness, particularly in the general [39] and working [40] population. Other noteworthy risk factors for common mental illness in the workforce include marital status and having a lower education [41].

While substantial research has explored the mental health of familial caregivers and its impact on child development, there is a notable gap in studies examining non-familial childcare providers. The mental health of all care providers, regardless of familial ties, is crucial to the development and well-being of children, as care provider-child interactions, particularly during the formative years, are essential for healthy child growth. Furthermore, there is limited evidence on modifiable psychosocial risk factors in the workplace that could alleviate mental health issues among childcare providers. Given the critical role of care provider-child interactions and the childcare environment in the first three years of life, this study aims to identify psychosocial factors associated with the mental health of preschool care providers in Kuala Lumpur.

## Methods

This cross-sectional study was conducted in Kuala Lumpur, Malaysia, from March to July 2019. Kuala Lumpur’s preschool areas were selected as statistics from the Social and Welfare Department showed it ranked second highest in child maltreatment cases, with 6.5% involving childcare providers to the child [17].

In Malaysia, the Ministry of Women, Family and Community Development (MWFCD) oversees childcare centers, while the Department of Social Welfare (JKM) regulates Early Childhood Care and Education (ECCE) programs [16, 42]. The JKM is responsible for the registration, inspection, and enforcement of centres to ensure children’s safety and development [42]. According to the Child Care Centre Act (1984), all institution-based centres must register, except home-based centres with fewer than four children [16, 42]. Requirements include licensing, adherence to health and safety standards, qualified staff, and maintaining appropriate caregiver-to-child ratios [43, 44]. Registered centres, known as Taman Asuhan Kanak-Kanak (TASKA), are classified into four types: home-based, workplace, community, and institution-based. Preschool settings vary in enrolment sizes, caregiver ratios, and educational approaches, with some accommodating as few as 10 children and others over 100. Caregiver-to-child ratios range from 5:1 to 15:1, especially in high-demand urban areas [12, 42, 44].

Utilizing Open Epi, the estimated sample size for this study was determined as *n* = 262, based on effect sizes from similar studies [45–47] (Supplementary Table 1). Thirty-six preschools were randomly selected from a sampling frame provided by the Kuala Lumpur Social and Welfare Department (138 preschools) for the study. All childcare providers aged 18 years and above working in preschools serving children under four were eligible to participate. Participants with acute psychiatric illness were excluded and referred for further assessment at the nearest clinic.

Written consent was obtained from each eligible respondent before enrolment. The data collection involved face-to-face interactions with the study subjects, utilizing standardized questionnaires covering sociodemographic details, the 5-item Santa Clara Strength of Religious Faith Questionnaire (SCSRFQ-5), the Center for Epidemiological Studies-Depression Scale (CES-D), and the Job Content Questionnaire (JCQ) (Supplementary Questionnaires). The survey took approximately 20 min to complete.

The sociodemographic questionnaire includes age, gender, ethnicity, marital status, education level, income status, family history of mental illness, chronic illness, and stressful life events. Participants indicate “yes” or “no” to major events causing emotional distress, such as assault, loss of a loved one, childhood abuse, serious marital problems, family problems, and injury or accidents. Participants were also asked whether they had received any pre-service training before enrolling as preschool providers (response options: 0 = not trained or 1 = trained). Data on total providers and number of children from the pre-school were also collected. A provider-to-child ratio (providers/number of children) was calculated based on the guidelines 1 to 5 for children < 3 years old.

Participants who answered “yes” to any life events were taken as positive for life events in the binary category [48]. For past medical illnesses, participants were asked about the presence of chronic disease for the past year or more. The positive answers were cross-checked with the participant’s home-based treatment card by the participant herself. They were guided by a list of chronic diseases adopted from a study by Kader Moideen et al. [39].

Participants assess the strength of religiosity using the 4-point Likert scale Santa Clara Strength of Religious Faith Questionnaire (SCSRFQ-5). Each of the five items is scored from 1 to 4 (1 = strongly disagree, 2 = disagree, 3 = agree, 4 = strongly agree) [49]. The cut-off point based on the sample median was adopted for the analysis [49]. The SCSRFQ-Malay has a Cronbach alpha coefficient of 0.84 [50] and current study yielded a Cronbach’s alpha coefficient of 0.83.

The CES-D, comprising 20 items in English and Malay, assessed subjects’ current depressive symptoms based on Radloff’s 1977 publication [51]. General population-based validation studies revealed a strong Cronbach alpha coefficient ranging from 0.75 to 0.90 for English and Malay language [51–53]. Participants reported depression-related symptoms from the past week, with each item scored from 0 to 3 (0 = Rarely or None of the Time, 1 = Some or Little of the Time, 2 = Moderately or Much of the Time, 3 = Most or Almost All the Time). All the items’ scores will be added up and total scores range from 0 to 60, and a score of 16 or higher indicates significant clinical depressive symptoms [28].

This study used English and Malay Job Content Questionnaires (JCQ) to measure job strain and psychological exposure. The Malay Version of the tool was validated with Cronbach’s alpha coefficients of 0.75 (decision latitude), 0.50 (job demand) and 0.84 (social support) [54]. The JCQ assesses psychosocial job domains, including skill discretion, demand, decision-making, supervisor support, co-worker support, and insecurity. Participants rated questions on a 4-point scale (1 = strongly disagree, 4 = strongly agree) on each domain of the JCQ, and reversed coding was employed for questions on job insecurity. The summation of scoring for each participant was based on the formula (Supplementary Table 2). The level of job strain is interpreted based on the Karasek Job Strain Model (Supplementary Fig. 1). Job demand components measure the psychological stressors concerning fulfilling workload, unexpected tasks, and job-related personal conflicts. Meanwhile, job decision latitude refers to the worker’s potential to control tasks and conduct during the working day [55]. The domain of support from supervisors and co-workers measures the level of social interaction among individuals working in the same preschool. Higher scores indicate better social support, which can help mitigate the negative effects of high job demands. The job security domain assesses employees’ perceived level of job security or insecurity regarding their current position, with higher scores reflecting greater perceived job insecurity [56].

The scoring disclosure was done privately in a separate room towards the end of the sessions. All the participants with high scores on CES-D were given a referral letter for further assessment at the nearest government clinics or hospitals. Participants received a confidential envelope with a referral letter, participation certificate, and contact details for additional counselling support through the local Befriender’s platform.

### Statistical analysis

Statistical analyses were conducted using IBM SPSS Statistics (version 24.0, IBM Corp LP, Armonk, NY, USA). The preliminary data exploration was conducted to identify the centrality, dispersion, normality, and missing data. Variables with < 5% missing data were included. Symptomatic depression was assessed using CES-D (scores ≥ 16 = depressed or < 16 = not depressed). For JCQ, variables that were included in the analysis were job strain, the variables were transformed to Low strain = 0, High Strain = 1. Each of the domains, supervisors, co-workers or insecurity, was dichotomized using a median score since their total score showed skewness. Effect modification first-order main effects of explanatory variables were checked using the likelihood-ratio test. The final model was assessed for assumption compliance, multicollinearity, and model fitness test. The rule of thumb is recommended by Paul et al. (2013) [57] was applied to ensure the effectiveness of the Hosmer-Lemeshow test. Model sensitivity was evaluated with the area under the receiving operating characteristics (AUROC) curve. A significance level of p-value < 0.05 was applied.

## Results

A total of 32 preschools have been visited. Two hundred forty-seven preschool care providers (*n* = 247) were recruited for this study, giving a response rate of 94.3%. Non-respondents were those of Chinese participants who refused to participate. The sociodemographic characteristics of the respondents are illustrated in Table 1. All the respondents were female, with a mean age of 32.1 years (SD = 12.0). The majority (55.5%) were less than 30, and 9.4% were more than 50 years old. Almost two-thirds of the respondents were from other states, followed by Selangor (21.9%) and Kuala Lumpur (14.2%). The respondents comprised Malay (70.4%), Chinese (11.4%), Indian (11.7%), and Other (6.5%). The other races were Bumiputera Sabah, Bumiputera Sarawak and a few non-Malaysians with valid legal documents. The majority of the preschool care providers were married (55.0%), followed by single (38.1%), divorcee (5.3%) and widowed (1.6%).

**Table 1:** Participant characteristic (*n* = 247)

Participant’s Characteristic	*n*	(%)
**Age (** *n* ** = 245)**		
**< 30** **30–39** **40–49** **≥ 50**	136 51 35 23	55.5 20.8 14.3 9.4
**Ethnicity**		
**Malay** **Chinese** **Indian** **Others**	174 28 29 16	70.4 11.4 11.7 6.5
**Marital Status**		
**Single** **Married** **Divorce/ Separated** **Widowed**	94 136 13 4	38.1 55.0 5.3 1.6
**Religion**		
**Muslim** **Christian** **Buddhist** **Hindu** **others**	178 25 16 23 5	72.1 10.1 6.5 9.3 2.0
**Religiosity (** *n* ** = 246)**		
**Low (< 17)** **High (≥ 17)**	94 152	38.2 61.8
**Education level**		
**None** **Primary** **Secondary** **certificate** **diploma and above**	1 6 112 3 125	0.4 2.4 45.3 1.3 50.6
**Monthly Income**		
**≤ RM 3860** **RM3861-RM 8318** **≥ RM 8319**	198 38 11	80.1 15.4 4.5
**Family History of mental illness (** *n* ** = 246)**		
**Yes** **No**	9 237	3.7 96.3
**Hx Chronic Illness (** *n* ** = 245)**		
**Yes** **No**	24 221	9.8 90.2
**Stressful life Event (** *n* ** = 246)**		
**Yes** **No**	94 152	38.2 61.8
**Training (** *n* ** = 244)**		
**Yes** **No**	226 18	92.6 7.4

Almost half of the care providers (50.6%) possessed a diploma or higher education, approximately 45.3% of the care providers completed secondary school, and a minority had education up to the primary school level. Based on the national socioeconomic classification for household income, most of the respondents (80.1%) earned below the low-income group (≤ RM 3860). The majority of the participants were Muslims, and a high proportion of them (61.8%) were found to have strong religious strength. Nine respondents (3.7%) reported that their 1st-degree or 2nd-degree family members had neurotic or psychotic-related mental illnesses. Twenty-four (9.8%) respondents reported having chronic illnesses such as hypertension, type 2 diabetes, asthma, and previous cancer history. One-third of the respondents (38.2%) had a history of stressful life events. The most frequently reported stressful life events were the loss of loved ones (24.0%), followed by severe chronic illness, serious family problems and serious financial problems.

Among the 247 female respondents, 28.7% of them fulfilled the depressive symptoms criteria rated by the CES-D. Based on the cut-off point (the sample median) for psychosocial job risk assessment, 40.9% had high job insecurity, 46.2% had low co-worker support, and 52.3% had low supervisor support. According to the Karasek’s formula, seventy-one participants (28.7%) perceived high job strain (Table 2).

**Table 2:** Psychosocial job characteristics

Psychosocial Job Characteristic	*n*	(%)
**CESD**		
**≥ 16**	71	28.7
**< 16**	176	71.3
**Providers to Child Ratio (** *n* ** = 241)**		
**> 1 in 4** **< 1 in 4**	138 103	57.3 42.7
**Job strain**		
**High** **Low**	71 176	28.7 71.3
**Job Insecurity**		
**Low** **High**	146 101	59.1 40.9
**Supervisor Support (** *n* ** = 243)**		
**Low** **High**	127 116	52.3 47.7
**Co-worker support**		
**Low** **High**	114 133	46.2 53.8

Table 3 demonstrates the relationship between sociodemographic and psychosocial job characteristics with depressive symptoms. The sociodemographic characteristics which had a significant relationship with depressive symptoms were marital status (OR = 2.73; 95% CI = 1.50, 4.93), monthly income (OR = 2.31; 95% CI = 1.02, 5.24), and stressful life event (OR = 1.91; 95% CI = 1.09, 3.35). The psychosocial job characteristics associated with depressive symptoms were high job strain (OR = 2.64; 95% CI = 1.47, 4.74), high job insecurity (OR = 3.40, 95% CI = 1.92, 6.04), and low supervisor support (OR = 1.63, 95% CI = 0.93, 2.85).

**Table 3:** Respondents characteristic in relation to depressive symptoms rated by CES-D (*n* = 247)

Participant’s Characteristic	Presence of Depression Symptoms CES-D ≥ 16 *n*(%)	Absence of Depression symptoms CES-D < 16 *n*(%)	χ^2^	Crude OR 95% CI		*P*-value
**Age**						
≥ 30	27(24.8)	82(75.2)		Reference		
< 30	44(32.3)	92(67.7)	1.690	1.45 (0.83, 2.55)		0.194
**Ethnicity**						
Non-Malay	16(21.9)	57(78.1)		Reference		
Malay	55(31.6)	119(68.4)	2.36	1.65 (0.87, 3.12)		0.127
**Marital Status**						
Non-Married	20(18.0)	91(82.0)		Reference		
Married	51(37.5)	85(62.5)	11.35	2.73 (1.50, 4.93)		< 0.001*
**Religion**						
Non-Muslim	56(31.5)	122(68.5)		Reference		
Muslim	15(21.7)	54(78.3)	2.29	1.652 (0.86, 3.18)		0.132
**Religiosity**						
≥ 17	38 (25.0)	114(75.0)		Reference		
< 17	33 (35.1)	61(63.9)	3.67	1.81 (0.98,3.32)		0.057
**Education level**						
Certificate and above	32(25.0)	96(75.0)		Reference		
Secondary and below	39(32.8)	80(67.2%)	1.82	1.46 (0.84, 2.54)		0.178
**Monthly Income**						
> RM3860	8 (16.7)	40 (83.3)		Reference		
≤ RM 3860	62(31.6)	134(68.4)	4.22	2.31 (1.02, 5.24)		0.044*
**Family History of mental illness**						
No	67(28.3)	170(71.7)		Reference		
Yes	4(44.4)	5(55.6)	1.11	2.03 (0.53,7.79)		0.293
**Hx Chronic Illness**						
No	66(29.9)	155(70.1%)		Reference		
Yes	5 (20.8)	19 (79.2)	0.86	0.62 (0.22, 1.73)		0.358
**Stressful life Event**						
No	36(23.7)	116(76.3)		Reference		
Yes	35(37.2)	59(62.8)	5.19	1.91 (1.09, 3.35)		0.023*
**Training**						
No	5(27.8)	13(72.2)		Reference		
Yes	66(29.2)	160(70.8)	0.02	1.07 (0.37, 3.13)		0.898
**Providers to Child Ratio**						
< 1 in 5	44(31.4)	96(68.6)		Reference		
> 1 in 5	27(25.2)	80(74.8)	1.14	0.74 (0.42, 1.29)		0.286
**Job strain**						
Low	40(22.7)	136(77.3)		Reference		
High	31(43.7)	40(56.3)	10.83	2.64 (1.47, 4.74)		0.001*
**Job Insecurity: total score**	7.0 (2.0)	6.0(2.0)	4343^a^	1.37 (1.16, 1.62)		0.001*
**Job Insecurity: Category**						
Low	27(18.5)	119(81.5)		Reference		
High	44(43.6)	57(56.4)	18.32	3.40 (1.92, 6.04)		< 0.001*
**Supervisor support: Total score**	1.0 (3.0)	2.0 (3.0)	5364^a^	0.17 (0.06, 0.30)		0.004*
**Supervisor support - Category**						
High	31(24.4)	96(75.6)		Reference		
Low	40(34.5)	76(65.5)	2.98	1.63 (0.93, 2.85)		0.085

*Significant at *P* < 0.05, a = Mann Whitney U

According to the Hosmer–Lemeshow criteria, 11 variables (p-value < 0.25) were included in the multivariable logistic regression model analysis. The final model contained five explanatory variables (Table 4). Married subjects were prone to depressive symptoms. Subjects who had a history of stressful life events, particularly for assault and marital issues, were at a 9-to-11-fold higher risk of getting depressive symptoms than the subjects without a history of stressful life events. Subjects with high job strain had a 2.33 times higher risk of developing depressive symptoms compared to the low job strain. Participants with high job insecurity had higher CES-D scores than those with low job insecurity. In contrast, support from supervisors was a protective factor against depressive symptoms.

**Table 4:** Risk factors of depressive symptoms as rated by CES-D (*n* = 247)

Factors	B	Crude OR (95% CI)	*P*-value	B	Adjusted OR (95% CI)	*P*-value
**Job Strain** Low High	Ref. 0.97	Ref. 2.64(1.47,4.74)	0.001	Ref. 0.84	Ref. 2.33(1.22, 4.44)	0.011*
**Supervisor Support**	-0.18	0.84 (0.74,0.94)	0.004	-0.13	0.88(0.77, 1.00)	0.048*
**Job Insecurity**	0.31	1.37 (1.16, 1.62)	< 0.001	0.27	1.29(1.07, 1.56)	0.006*
**Marital Status** Non-married Married	Ref. 1.11	Ref 3.04(1.60,5.78)	< 0.001	Ref 0.79	Ref 2.50(1.26, 4.96)	0.009*
**SL – Assault** No Yes	Ref. 1.18	Ref 3.24(0.84,12.43)	0.087	Ref 1.77	Ref 8.96(1.66, 48.45)	0.011*
**SL- Marital Issues** No Yes	Ref. 2.25	Ref 9.46(1.92, 46.74)	0.006	Ref 2.15	Ref 10.67(2.30,49.36)	0.002*

*Significant at *P* < 0.05

Model Development: The Hosmer-Lemeshow strategy 4, entry criterion of *P*-values < 0.25 backwards in purposive variables exclusion with *P*-value > 0.05 guided by the likelihood-ratio test. All the models were assessed for good model fitting (AUC: 0.770, R²N: 0.2564, VIF < 5 classification Table 0.895), SL – Stressful Life Event, Ref. – Reference.

## Discussions

This study provides one of the first studies to explore the prevalence of depressive symptoms among preschool care providers and the associated risk factors in Kuala Lumpur. The current study has revealed a high prevalence of depressive symptoms (28.7%). At the global level, few related studies on depression were conducted in the United States of America, targeting female educators from the state-funded early childhood programme [47]. By adopting a similar self-reported instrument (CES-D), the prevalence rates of clinically significant depressive symptoms reported were estimated as 9.4–24% [28, 47]. The lower prevalence rates found in the current study may be attributed to America’s more structured and well-established education system, whereby the workers may have better resources to handle the children. Besides, the difference may be due to different work cultures in other countries despite using the same instrument as in Malaysia.

As conceptualised by Raina et al. for caregiving, the care providers’ psychological health outcomes were determined by a few factors, which include personal background factors and work-related strain [58]. Married care providers exhibited significantly higher odds of developing depression compared to their unmarried counterparts. These findings were contradicted by substantial evidence from a systematic review with meta-analysis confirming that widowhood is a predictor for depression among the working population [59]. Another study from China showed no significant findings between marital status and mental health issues among preschool teachers [60]. Working women often have additional demands at home and trying to accomplish both roles may increase their stress levels [61]. Therefore, demands from female teachers’ personal lives, including marital issues and home, may be a source of increased stress levels [61].

Stressful life events were found to be a risk factor for developing depressive symptoms in this studied population in the final analysis. The most frequent life events found among the care providers were childhood bullying or abuse, loss of loved ones, serious family issues and serious financial issues. Out of these events, serious family issues were statistically associated with depression. These findings were consistent with other studies done locally and in Canada [39, 62, 63]. A national survey in Canada found that among the adult working population with a history of adverse life events were 1.9 times (OR = 1.90; 95% CI = 1.51, 2.40) higher risk of developing depression, and this is consistent with the findings in this study [63]. Another two local cross-sectional studies among the general population have stratified and analysed their results according to individual life events. Among the significant events were serious issues at the workplace, serious issues with friends and neighbours, serious marital problems, financial issues, and family issues [39, 62]. This study reinforces the link between stressful life events, especially serious family issues, and the onset of depressive symptoms among care providers in Malaysia. Relationship and marital difficulties are key stressors that significantly contribute to mental health challenges. Women experiencing physical violence, whether from intimate partners or within family settings, are particularly vulnerable, as physical abuse often coincides with psychological abuse, creating an environment that diminishes self-esteem and coping capacity [37, 64, 65]. Malaysian childcare providers face additional stress as they balance professional duties with personal challenges. Intimate partner violence (IPV) is a significant issue, with emotional abuse being the most prevalent form, often coupled with physical violence [66]. The National Health and Morbidity Survey reported that nearly 500,000 Malaysian women have experienced some form of IPV, mainly emotional or psychological abuse [67]. Cultural norms in Malaysia may exacerbate IPV by condoning violence and leading to underreporting, preventing women from seeking help. Empowerment and advocacy programs are crucial for providing resources like legal aid and counselling while raising awareness about women’s rights. Future research should consider utilizing specific IPV assessment tools, such as Sect. 7 (Experiences of Partner Violence) from the WHO Multi-country Study on Women’s Health and Life Events Questionnaire [68, 69]. This approach may more accurately evaluate IPV prevalence and its impact on mental health. Such data could inform the development of targeted interventions aimed at addressing the root causes of IPV and improving mental health outcomes among affected women, including those in caregiving roles.

For the final model of multivariable analyses, job strain was found to be significantly related to depressive symptoms rated by CES-D. Job strain is a well-recognized risk factor for depression and has been proven by substantial cross-sectional studies, longitudinal studies, systematic reviews, and meta-analyses. A large cohort study conducted by Stansfield et al. in 2012 reported that repeated high job strain on a few occasions increases the risk of major depression (AOR = 2.19; 95% CI = 1.48, 3.26) [70].

Job insecurity was associated with depressive symptoms as well. Workplace conditions have changed tremendously. These changes lead to more job demand; thus, workers would compete to work harder to keep pace. Consequently, it would increase workplace stress and cause employees to feel insecure about their jobs. Eventually, it will add to the job strain and contribute to the occurrence of mental illness. Therefore, a supportive supervisor is a positive factor that may play a crucial role in reducing the job stress faced by childcare providers. A Canadian community-based study revealed that higher job security and social support give rise to flourishing mental health [71]. A recent longitudinal study has revealed that high job demand with low social support in the long term increases the risk of depression [35]. According to the 2012 preschool regulations, each childcare provider must attend and pass the early childcare course. The Social and Welfare Department should consider implementing a managerial skills course for supervisors to enhance communication and better support the needs of childcare workers under their supervision. Expected associations with religiosity, family history, and history of chronic illness were not observed in this study, which is likely attributed to the smaller sample size and relatively high proportion of young workers. This study has constituted fewer positive cases with the respective underlying exposures for inferential analysis.

This pioneering study explores the mental health state of Malaysian preschool care providers using a local language-validated tool. It yields insights to inform policy improvements for their working environment. The findings serve as a stepping stone for future large-scale studies to enhance the generalizability and address caregiver mental health issues.

This study has several limitations. Its cross-sectional design hinders establishing true causal relationships between outcomes and exposure. Bidirectional relationships among some variables remain uncertain without temporal relationships in a cross-sectional study. Pre-existing depression in caregivers may influence perceptions of job strain [35]. Although the CES-D was a validated tool with good reliability, it could not replace the gold standard tools like the Structured Clinical Interview for DSM-IV (SCID) and confirmation of diagnosis by a clinical physician [72]. However, the cost of diagnostic tools poses a challenge in research in low- and middle-income countries (LMIC). Recall biases may affect responses, particularly for symptoms present over the past two weeks. Efforts were made to mitigate biases through participant briefings. The study tools for this study are self-reporting questionnaires and potential for social desirability bias. Mental disorders are a sensitive condition to be reported by the employee for fear of losing their employment. Therefore, the participants were informed and reassured on the confidentiality and the level of anonymity of the survey. This includes the generation and presentation the final data in a collective manner [72]. There was a notable refusal rate among Chinese providers, consistent with patterns observed in other studies in the country. Therefore, future research should delve into the reasons behind these refusals. Consequently, caution is advised when interpreting the study’s findings.

## Conclusion

High prevalence of depressive symptoms among Kuala Lumpur’s preschool care providers is attributed to job strain and insecurity. To address this, the Social and Welfare Department could advocate for supportive supervision, including skill training for supervisors to create a positive mental health environment. To enhance overall risk assessment, the public health department could integrate mental health screening into preschool inspections, focusing on individual risk factors. Collaborative efforts between Public Health and Social and Welfare Department stakeholders are crucial for identifying gaps and developing strategies to enhance workers’ mental health for children’s safety.

## Electronic supplementary material

Below is the link to the electronic supplementary material.

Supplementary Material: Additional file 1 - STROBE Statement—Checklist of items that should be included in reports of cross-sectional studies

## Data Availability

Due to the sensitivity of the area of study and for data protection purposes, the data used in this study cannot be accessed publicly. However, researchers who wish to obtain the dataset for research purposes, they can directly email to the corresponding author (dr.estherwong@moh.gov.my) upon reasonable request and with the necessary permission.

## Abbreviations

AOR: Adjusted Odd ratio
AUC: Area under curve
AUROC: Area under the receiving operating characteristics
CES-D: Center for Epidemiologic Studies Depression Scale
CI: Confidence interval
JCQ: Job-Content-Questionnaires
LMIC: Low- and middle-income countries
OR: Odd ratio
Ref.: Reference
SCID: Structured Clinical Interview for DSM-IV
SCSRFQ-5: 5-item Santa Clara Strength of Religious Faith Questionnaire
SD: Standard deviation
SL: Stressful life event
VIF: Variance inflation factor
