# Supplementary material for: Psychosocial risks and mental health of preschool care providers in Kuala Lumpur, Malaysia: a cross-sectional study
Source: BMC Psychol. 2025 Jan 9;13:25. doi: 10.1186/s40359-024-02279-3 (PMC11720848; doi:10.1186/s40359-024-02279-3)

**Title:** Psychosocial Risks and Mental Health of Preschool Care Providers in Kuala Lumpur,

Malaysia: A cross-sectional study.

**Journal:** Social Psychiatry and Psychiatric Epidemiology, The International Journal for Research in Social and Genetic Epidemiology and Mental Health Services

**Authors:** Tham Sin Wan^1^*, Wong Min Fui @ Esther Wong^2^*, Maslinor Binti Ismail^3^ and Noriklil Bukhary Binti Ismail Bukhary^4^

**Affiliations: Full information for each author**

^1^Centre for Burden of Disease Research, Institute for Public Health, National Institutes of Health, Malaysia

^2^Centre for Health Policy Research, Institute for Health Systems Research, National Institute of Health, Malaysia
^3^Department of Social and Preventive Medicine, Faculty of Medicine, University Malaya, Malaysia
^4^Health office of Lembah Pantai District, Health Department of Kuala Lumpur and Putrajaya, Ministry of Health, Malaysia
 *****Tham Sin Wan and Wong Min Fui @ Esther Wong contributed equally to this work.

**Correspondence**:

Wong Min Fui @ Esther Wong, MD

Centre for Health Policy Research, Institute of Health System Research, National Institute of Health, Malaysia

Blok B2, Kompleks Institut Kesihatan Negara (NIH)

No.1, Jalan Setia Murni U13/52, Seksyen U13 Setia Alam, 40170 Shah Alam, Selangor.

E-mail: dr.estherwong@moh.gov.my

**Supplementary Materials**

1. Supplementary Table 1: Sample Size Calculation
2. Supplementary Table 2: The formula used for each psychological work exposure variable are listed below:
3. Supplementary Figure 1: Karasek Job Strain Model
4. Supplementary Questionnaires

# Supplementary Table 1: Sample Size Calculation

| Objectives |  | Calculated Sample Size |
| --- | --- | --- |
| Prevalence |  | 213 |
| Risk factors for Mental Disorders |  | 262 |
| Job strain and Mental Disorders |  | 228 |

**Supplementary Table 2**: The formula used for each psychological work exposure variable are listed below:

| **Domains** | **Formula** | **Score** |
| --- | --- | --- |
| Job skill discretion | [s1 + s3 + s5 + s7 + s9 + 5 - s2] x 2 | 12-48 |
| Job demands | 3(s10 + s11) + 2(15 - s12 - s13 - s14) | 12-48 |
| Job decision-making authority | [S4+s8+(5-s6)]x4 | 12-48 |
| Job insecurity = | s24 + s26 + 5 - s15. | 3-12 |
| Co-worker support | s16 + s17 + s18 + s19. | 4-16 |
| Supervisor support | s20 + s21 + s22 + s23 | 4-16 |
| Job decision latitude | skill discretion + decision-making authority | 24-96 |

Job decision latitude consists of skill discretion and decision-making authority and each variable will be calculated for the mean and classified accordingly.

The Psychological work exposure is defined as below:

1. A score > sample median on job demands: denote high job demand
2. A score < sample median on job decision latitude :Denote low Job control
3. A score < sample median on co-worker support: low support from co-worker
4. A score < sample median supervisor support : low supervisor support
5. A score >sample median on job security: Insecurity

The quadrant term formulation will be adopted to calculate for the job strain.

# Supplementary Figure 1 Karasek Job Strain Model


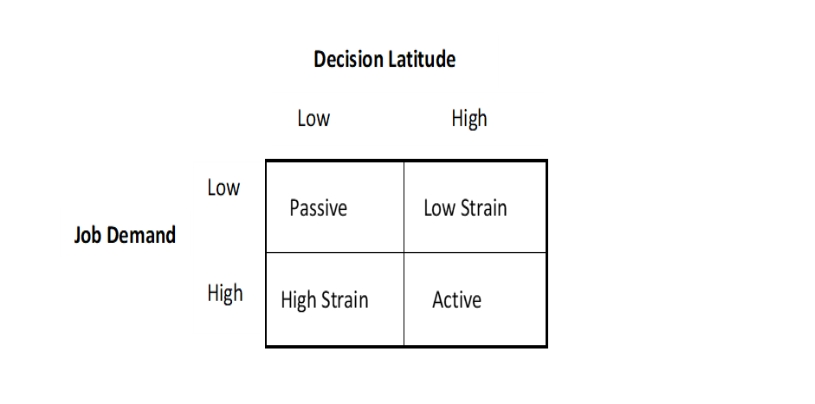


# Supplementary Questionnaires


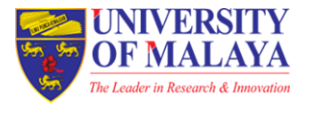


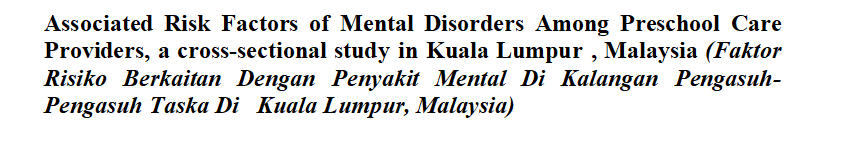

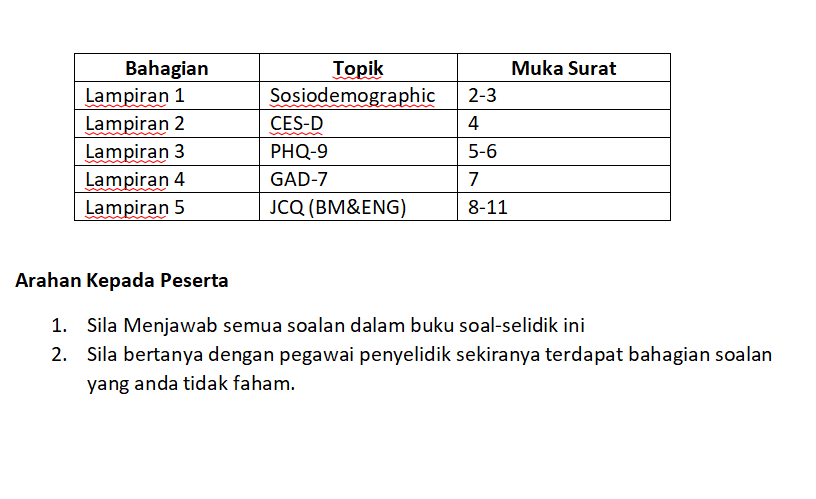

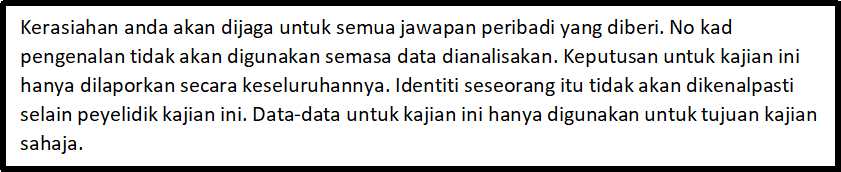


**Lampiran 1: Sociodemographic Questionnaire/ *Borang Soal Selidik***

**To be filled by participant / *Untuk diisi oleh peserta***

**Instruction / *Arahan*:** Please tick **(√)** **ONE** most appropriate answer.

***Sila tandakan (√) SATU pilihan jawapan yang paling tepat.***

| **No.** | **Question / *Soalan*** | **Answer / *Jawapan*** | **Remarks/ *Catatan*** | |
| --- | --- | --- | --- | --- |
| 1. | Gender / ***Jantina*** | 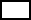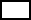 Male / **lelaki** Female / **Perempuan** | |  |
|  |  |  | |  |
| 2. | Place of Origin/***Tempat Berasal*** |  | |  |
|  |  |  | |  |
| 3. | Year of birth / **Tahun lahir** | **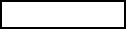** (example / *contoh*: 1957) | |  |
|  |  |  | |  |
| 4. | Race / ***Bangsa*** | 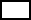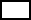 Malay / ***Melayu*** Indian / ***India*** | |  |
|  |  | 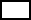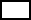 Chinese / ***Cina*** Others / ***Lain-lain*** | |  |
|  |  |  | |  |
| 5. | Marital Status/ ***Status Perkahwinan*** | 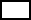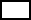 Single/**Bujang** Married/**Kahwin** | |  |
|  |  | 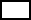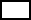 Divorced/**Bercerai** Separated/**Berpisah** | |  |
|  |  | 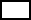 Widowed/Balu | |  |
|  |  |  | |  |
| 6. | Highest education level / ***Tahap pendidikan tertinggi*** | 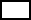 No formal education/ ***Tiada Pendidikan formal*** | |  |
|  |  | 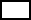 Primary school / ***Sekolah rendah*** | |  |
|  |  | 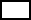 Secondary school / ***Sekolah menengah*** | |  |
|  |  | 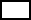 Diploma and above / ***Diploma ke atas*** | |  |
|  |  |  | |  |
| 7. | Monthly household | 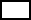 **B 40** Less than RM2,500 / ***Kurang dari RM2500*** | |  |
|  | income / ***Pendapatan*** | 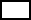 **M 40** RM2,5001 – RM 11,000 | |  |
|  |  | 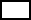 **T 20** RM 11,001 / ***Lebih dari RM 11,001*** | |  |
|  |  |  | |  |
| 8. | Religion/ | 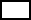 Muslim/ ***Islam*** | |  |
|  | ***Agama*** | 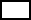 Christianity/***Kristian*** | |  |
|  |  | 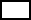 Buddhism/ ***Buddist*** | |  |
|  |  | 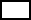 Hindu/ ***Hindu*** | |  |
|  |  | 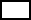 Lain-lain : ____________________________ | |  |
|  |  |  | |  |
| 9. | Strength of religious faith*/* ***Kekuatan kepercayaan dan keagamaan*** | 1 = strongly disagree 2 = disagree 3 = agree 4 = strongly agree | |  |
|  |  | - I pray daily/ ***Saya sembahyang setiap hari*** | |  |
|  |  | - I look to my faith as providing meaning and purpose in   my life/***Pegangan agama saya memberi***  ***makna dan tujuan dalam hidup saya***. | |  |
|  |  | - I consider myself active in my religious organization/   ***Saya bergerak aktif dalam organisasi keagamaan.*** | |  |
|  |  | - I enjoy being around others who share my faith/ ***saya***   ***suka dikelilingi oleh individu berkongsi pegangan***  ***agama yang sama*** | |  |
|  |  | - My faith impacts many of my decisions/ ***Pegangan***   ***agama saya mempengaruhi banyak keputusan yang saya buat*** | |  |
|  |  | 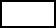 **Total score** ***(5 : low in religious faith 20 high in religious faith )*** | |  |
|  |  | (*Adapted from : Santa Clara Strength of Religious Faith Questionnaire Plante et al (1997) copyright: Public Domain*) | |  |
|  |  |  | |  |
| 10. | Any family members having mental illness/ ***Sejarah ahli keluarga adanya penyakit sakit jiwa*** | - Yes/ ***Ya*** | |  |
|  |  | - No/ ***Tidak*** | |  |
|  |  | Relatioship?/ ***Hubungan ?***_______________________ | |  |
|  |  |  | |  |
|  |  |  | |  |
|  |  |  | |  |
| 11. | Any past medical Illness/***Sejarah penyakit Kronik*** | 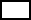 No/***Tidak*** | |  |
|  |  | 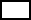 Yes/ ***Ya*** Nyatakan**_______________________** | |  |
|  |  | Please state/ ***Tolong nyatakan:*** | |  |
|  |  | *Years since diagnosed /*  ***Tempoh sejak didiagnosa dengan penyakit (dalam***  ***tahun):_____________*** | |  |
|  |  | Treatment/***Rawatan:*** **______________________** | |  |
|  |  |  | |  |
| 12. | Stressful Life Event/***Pengalaman Perit dalam Hidup*** | 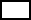 No/ ***Tidak*** | |  |
|  |  |  | |  |
|  |  | 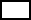 if Yes/ ***Ya***,  please choose your answer/***sila pilih jawapan anda:*** | |  |
|  |  | 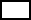 1.Assault/ ***serangan (Physical or sexual/ fizkal atau***  ***sexual)*** | |  |
|  |  | 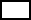 2.Serious chronic Illness/ ***Penyakit yang serius dan***  ***Kronik (Seperti Kencing manis atau Darah Tinggi)*** | |  |
|  |  | 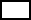 3.Abuse or bully during childhood/ ***didera dan dibuli***  ***semasa zaman kanak- kanak*** | |  |
|  |  | 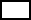 4.Serious injury due to accident/ ***kecederaan***  ***disebabkan oleh kemalangan*** | |  |
|  | **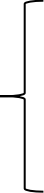Both Parents** | 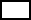 5.Being an Orphan before the age 10/***anak yatim***  ***sebelum umur 10 tahun*** | |  |
|  | **One of the parents and other relatives** | 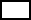 6.Lost of love ones / ***kehilangan orang tersayang*** | |  |
|  |  | 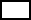 7.Serious marital problem/ ***masalah rumah tangga***  ***yang serius*** | |  |
|  |  | 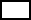 8.Serious family problem/ ***masalah keluarga yang***  ***serius*** | |  |
|  | 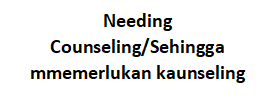 | 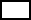 9.Serious financial problem/ ***masalah kewangan yang***  ***Serius(Bankrupsy / Bankrup)*** | |  |
|  |  | 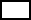 10.Serious housing problem (poor relationship with  Neigbourhood or high crime rate in the neigbourhood)  /***masalah tempat kediaman yang serius (masalah***  ***dengan jiran atau kadar jenayah yang tinggi)*** | |  |
|  |  | 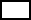 11.Serious problem at work (problem with superior)/  ***masalah di tempat kerja (masalah dengan majikan)*** | |  |
|  |  | 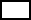 12.Lost of Job/ ***kehilangan pekerjaan*** | |  |
|  |  | 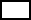 13.Court case / ***Kes mahkamah*** | |  |
| 13. | Information of the preschool***/ Maklumat berkaitan dengan taska*** | Trained**/ *dilatih***: yes/**ya** no/**tidak** | |  |
|  |  | **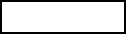**No. Care providers/ ***Bilangan Penjaga*** | |  |
|  |  | **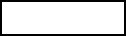**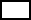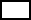No of Children/  ***Bilangan Kanak-kanak di bawah 4 tahun*** | |  |
|  |  |  | |  |

**LAMPIRAN 2: (CES-D):** Centre of EPusat untuk Kajian Epidemiologi Skala Kemurungan

**Arahan: Di bawah adalah senarai sebahagian perkara yang anda mungkin rasa atau lakukan.**

**Please tick (√) ONE most appropriate answer.** Tandakan (√) pada kenyataan yang paling tepat dengan diri anda.

| **Sepanjang minggu lepas** | **Jarang / tiada** | **Kadang-kadang**  **(1-2 hari)** | **Kerapkali**  **(3-4 hari)** | **Pada setiap masa (5-7 hari)** |
| --- | --- | --- | --- | --- |
| **1.I was bothered by things that usually don’t bother me**  *Fikiran saya diganggu oleh hal yang selalunya tidak menggangu saya* |  |  |  |  |
| **2.I did not feel like eating; my appetite was poor**  *Saya tiada selera untuk makan* |  |  |  |  |
| **3.I felt that I could not shake off the blues even with help from my family or friends**  *Saya rasa saya tidak dapat menghapuskan perasaan tertekan walaupun dengan bantuan kawan-kawan saya* |  |  |  |  |
| **4.I felt that I was just as good as other people**  *Saya rasa saya sebaik orang lain* |  |  |  |  |
| **5.I had trouble keeping my mind on what I was doing***.*  *Saya mempunyai masalah untuk menumpukan perhatian kepada kerja yang saya lakukan* |  |  |  |  |
| **6.I felt depressed.**  *Saya rasa tertekan* |  |  |  |  |
| **7.I felt that everything I did was an effort**  *Saya rasa semua yang saya lakukan adalah satu usaha* |  |  |  |  |
| **8.I felt hopeful about the future.**  *Saya rasa mempunyai harapan yang baik untuk masa depan saya* |  |  |  |  |
| **9.I thought my life had been a failure**  *Saya fikir hidup saya telah mengalami kegagalan* |  |  |  |  |
| **10.I felt fearful.**  *Saya merasa sangat takut* |  |  |  |  |
| **11.My sleep was restless**  *Tidur saya terganggu* |  |  |  |  |
| **12.I was happy**  *Saya gembira* |  |  |  |  |
| **13.I talked less than usual**  *Saya bercakap kurang berbanding kebiasaannya* |  |  |  |  |
| **14.I felt lonely.**  *Saya berasa kesunyian* |  |  |  |  |
| **15.People were unfriendly**  *Orang di sekeliling saya tidak mesra* |  |  |  |  |
| **16.I enjoyed life.**  *Saya menikmati hidup saya* |  |  |  |  |
| **17.I had crying spells.**  *Saya rasa seolah-olah ingin menangis***.** |  |  |  |  |
| **18.I felt sad.**  *Saya berasa sedih* |  |  |  |  |
| **19.I felt that people dislike me.**  *Saya rasa orang lain tidak sukakan saya* |  |  |  |  |
| **20.I could not get “going.”**  *Saya tidak dapat meneruskan hidup* |  |  |  |  |

**Lampiran 3: PATIENT HEALTH QUESTIONNAIRE (PHQ-9) (MALAY VERSION)**

Dalam tempoh 2 minggu yang lepas, berapa kerapkali anda terganggu oleh masalah berikut? / Over the last 2 weeks, how often have you been bothered by any of the following problems?

| **No.** | **SOALAN** | **SKALA** | **SKOR** |
| --- | --- | --- | --- |
| 1. | **Sedikit minat atau sedikit keseronokan dalam melakukan kerja-kerja.**  Little interest or pleasure in doing things. | **Tidak pernah sama sekali** / Not at all | 0 |
|  |  | Beberapa hari / Several days | 1 |
|  |  | **Lebih dari seminggu** / More than half the days | 2 |
|  |  | **Hampir setiap hari** / Nearly everyday | 3 |
|  |  |  |  |
| 2. | **Merasa murung, sedih atau tiada harapan.**  Feeling down, depressed or hopeless. | **Tidak pernah sama sekali** / Not at all 0 | 0 |
|  |  | **Beberapa hari** / Several days | 1 |
|  |  | **Lebih dari seminggu** / More than half the days | 2 |
|  |  | **Hampir setiap hari** / Nearly everyday | 3 |
|  |  |  |  |
| 3. | **Masalah hendak tidur / semasa tidur, tidur terlalu banyak.**  Trouble falling / staying asleep, sleeping too much | **Tidak pernah sama sekali** / Not at all 0 | 0 |
|  |  | **Beberapa hari** / Several days | 1 |
|  |  | **Lebih dari seminggu** / More than half the days | 2 |
|  |  | **Hampir setiap hari** / Nearly everyday | 3 |
|  |  |  |  |
| 4 | **Merasa letih atau kurang bertenaga**.  Feeling tired or having little energy. | **Tidak pernah sama sekali** / Not at all | 0 |
|  |  | **Beberapa hari** / Several days | 1 |
|  |  | **Lebih dari seminggu** / More than half the days | 2 |
|  |  | **Hampir setiap hari** / Nearly everyday | 3 |
|  |  |  |  |
| 5 | **Kurang selera atau terlalu banyak makan**.  Poor appetite or over eating. | **Tidak pernah sama sekali** / Not at all | 0 |
|  |  | **Beberapa hari** / Several days | 1 |
|  |  | **Lebih dari seminggu** / More than half the days | 2 |
|  |  | **Hampir setiap hari** / Nearly everyday | 3 |
|  |  |  |  |
| 6 | **Mempunyai perasaan buruk terhadap diri sendiri – ataupun merasa gagal terhadap diri sendiri ataupun menghampakan diri atau keluarga.**  Feeling bad about yourself – or that you are a failure or have let yourself or your family down? | **Tidak pernah sama sekali** / Not at all | 0 |
|  |  | **Beberapa hari** / Several days | 1 |
|  |  | **Lebih dari seminggu** / More than half the days | 2 |
|  |  | **Hampir setiap hari** / Nearly everyday | 3 |
|  |  |  |  |
| 7 | **Masalah menumpukan perhatian terhadap perkara-perkara seperti membaca suratkhabar atau menonton televisyen**  Trouble concentrating on things, such as reading the newspaper or watching television. | **Tidak pernah sama sekali** / Not at all | 0 |
|  |  | **Beberapa hari** / Several days | 1 |
|  |  | **Lebih dari seminggu** / More than half the days | 2 |
|  |  | **Hampir setiap hari** / Nearly everyday | 3 |
| 8 | **Bergerak atau bercakap dengan terlalu lambat sehingga disedari oleh orang lain. Ataupun bertentangan – terlalu resah atau gelisah sehingga anda bergerak lebih dari biasa**.  Moving or speaking so slowly that other people could have noticed. | **Tidak pernah sama sekali** / Not at all | 0 |
|  |  | **Beberapa hari** / Several days | 1 |
|  |  | **Lebih dari seminggu** / More than half the days | 2 |
|  |  | **Hampir setiap hari** / Nearly everyday | 3 |
|  |  |  |  |
| 9 | **Berfikiran bahawa lebih elok jika anda telah mati atau inginmencederakan diri anda dalam sesuatu cara.**  Thoughts that you would be better off dead or of hurting yourself in some way. | **Tidak pernah sama sekali** / Not at all | 0 |
|  |  | **Beberapa hari** / Several days | 1 |
|  |  | **Lebih dari seminggu** / More than half the days | 2 |
|  |  | **Hampir setiap hari** / Nearly everyday | 3 |

|  |  |  |  |
| --- | --- | --- | --- |
|  |  |  |  |
|  |  |  |  |
|  |  |  |  |
|  |  |  |  |
|  |  |  |  |
|  |  |  |  |

**Lampiran 4 : JOB CONTENT QUESTIONNAIRE(BM)**

Soal-selidik penilaian risiko pendedahan terhadap hazad psikologi di tempat kerja

**Bulatkan jawapan yang betul.**

|  | | | | | **Sangat tidak setuju** | **Tidak setuju** | | | **Setuju** | | | **Sangat setuju** |
| --- | --- | --- | --- | --- | --- | --- | --- | --- | --- | --- | --- | --- |
| 1. Pekerjaan saya memerlukan saya mempelajari perkara baru. | | | | | 1 | 2 | | | 3 | | | 4 |
| 1. Pekerjaan saya melibatkan kerja yang berulang-ulang. | | | | | 1 | 2 | | | 3 | | | 4 |
| 1. Pekerjaan saya memerlukan kreativiti. | | | | | 1 | 2 | | | 3 | | | 4 |
| 1. Pekerjaan saya membenarkan saya membuat keputusan sendiri. | | | | | 1 | 2 | | | 3 | | | 4 |
| 1. Pekerjaan saya memerlukan kemahiran yang tinggi. | | | | | 1 | 2 | | | 3 | | | 4 |
| 1. Semasa bekerja, saya diberi banyak kebebasan untuk membuat keputusan sendiri. | | | | | 1 | 2 | | | 3 | | | 4 |
| 1. Semasa bekerja, saya berupaya melakukan berbagai perkara yang berbeza-beza. | | | | | 1 | 2 | | | 3 | | | 4 |
| 1. Saya mempunyai banyak hak untuk menentukan pekerjaan saya. | | | | | 1 | 2 | | | 3 | | | 4 |
| 1. Saya berpeluang untuk mengembangkan kebolehan saya. | | | | | 1 | 2 | | | 3 | | | 4 |
| 1. Pekerjaan saya memerlukan saya untuk bekerja dengan sangat pantas. | | | | | 1 | 2 | | | 3 | | | 4 |
| 1. Pekerjaan saya memerlukan saya bekerja bersungguh-sungguh. | | | | | 1 | 2 | | | 3 | | | 4 |
| 1. Saya tidak diminta / disuruh untuk melakukan kerja-kerja secara berlebihan. | | | | | 1 | 2 | | | 3 | | | 4 |
| 1. Saya mempunyai masa yang cukup untuk menyiapkan kerja saya. | | | | | 1 | 2 | | | 3 | | | 4 |
| 1. Saya bebas daripada tekanan-tekanan yang dibuat oleh orang lain. | | | | | 1 | 2 | | | 3 | | | 4 |
| 1. Pekerjaan saya dijamin baik. | | | | | 1 | 2 | | | 3 | | | 4 |
| 1. Rakan-rakan sekerja saya berkemampuan dalam melakukan kerja mereka. | | | | | 1 | 2 | | | 3 | | | 4 |
| 1. Rakan-rakan sekerja mengambil berat tentang saya. | | | | | 1 | 2 | | | 3 | | | 4 |
| 1. Rakan-rakan sekerja saya adalah peramah. | | | | | 1 | 2 | | | 3 | | | 4 |
| 1. Rakan-rakan sekerja saya membantu bagi memastikan kerja-kerja disiapkan. | | | | | 1 | 2 | | | 3 | | | 4 |
| 1. Penyelia saya mengambil berat mengenai kebajikan orang bawahannya. | | | | | 1 | 2 | | | 3 | | | 4 |
| 1. Penyelia saya memberikan perhatian terhadap apa yang saya katakan. | | | | | 1 | 2 | | | 3 | | | 4 |
| 1. Penyelia memberi bantuan dalam memastikan kerja-kerja saya dapat disiapkan. | | | | | 1 | 2 | | | 3 | | | 4 |
| 1. Penyelia saya berjaya mengajak orang lain bekerja bersama-sama. | | | | | 1 | 2 | | | 3 | | | 4 |
|  | Tetap dan stabil | | Ber  musim | | Kerap  tergendala | | | Bermusim dan kerap tergendala | | | Lain-lain | |
| 1. Berapa stabilkah kerja anda? | 1 | | 2 | | 3 | | | 4 | | | 5 | |
|  | Tidak pernah | | Sekali | | Lebih dari sekali | | | Sentiasa | | | Diberhentikan | |
| 1. Dalam tempoh setahun yang lepas, berapa kerap anda berdepan dengan masalah kehilangan pekerjaan? | 1 | | 2 | | 3 | | | 4 | | | 5 | |
|  | | Tidak mungkin | | Sedikit kemungkinan | | | Berkemungkinan | | | Berkemungkinan besar | | |
| 1. Kadangkalaseseorang itu kehilangan pekerjaan tetap mereka. Adakah kemungkinan anda akan kehilangan pekerjaan anda sekarang dalam beberapa tahun lagi? | | 1 | | 2 | | | 3 | | | 4 | | |

©Copyright by Robert Karasek JCQ Center. All rights reserved. No distribution without permission from The JCQ Center by Director Robert Karasek

**Lampiran 4 : JOB CONTENT QUESTIONNAIRE(ENGLISH)**

Risk assessment on exposure to psychological hazard at workplace.

**Please circle the correct answer.**

|  | | | | strongly disagree | | disagree | agree | | strongly agree |
| --- | --- | --- | --- | --- | --- | --- | --- | --- | --- |
| 1. My job requires that I learn new things | | | | 1 | | 2 | 3 | | 4 |
| 1. My job involves a lot of repetitive work | | | | 1 | | 2 | 3 | | 4 |
| 1. My job requires me to be creative | | | | 1 | | 2 | 3 | | 4 |
| 1. My job allows me to make a lot of decisions on my own | | | | 1 | | 2 | 3 | | 4 |
| 1. My job requires a high level of skill | | | | 1 | | 2 | 3 | | 4 |
| 1. On my job, I am given a lot of freedom to decide how I do my work | | | | 1 | | 2 | 3 | | 4 |
| 1. I get to do a variety of things on my job | | | | 1 | | 2 | 3 | | 4 |
| 1. I have a lot to say about what happens on my job | | | | 1 | | 2 | 3 | | 4 |
| 1. I have an opportunity to develop my own special abilities | | | | 1 | | 2 | 3 | | 4 |
| 1. My job requires working very fast | | | | 1 | | 2 | 3 | | 4 |
| 1. My job requires working very hard | | | | 1 | | 2 | 3 | | 4 |
| 1. I am not asked to do an excessive amount of work | | | | 1 | | 2 | 3 | | 4 |
| 1. I have enough time to get the job done | | | | 1 | | 2 | 3 | | 4 |
| 1. I am free from conflicting demands others make | | | | 1 | | 2 | 3 | | 4 |
| 1. My job security is good | | | | 1 | | 2 | 3 | | 4 |
| 1. People I work with are competent in doing their jobs | | | | 1 | | 2 | 3 | | 4 |
| 1. People I work with take a personal interest in me | | | | 1 | | 2 | 3 | | 4 |
| 1. People I work with are friendly | | | | 1 | | 2 | 3 | | 4 |
| 1. People I work with are helpful in getting the job done | | | | 1 | | 2 | 3 | | 4 |
| 1. My supervisor is concerned about the welfare of those under him | | | | 1 | | 2 | 3 | | 4 |
| 1. My supervisor pays attention to what you are saying | | | | 1 | | 2 | 3 | | 4 |
| 1. My supervisor is helpful in getting the job done | | | | 1 | | 2 | 3 | | 4 |
| 1. My supervisor is successful in getting people to work together | | | | 1 | | 2 | 3 | | 4 |
|  | regular and steady | seasonal | | frequent layoffs | | | both seasonal and frequent layoffs | | other |
| 24. How steady is your work? | 1 | 2 | | 3 | | | 4 | | 5 |
|  | never | faced possibility once | | faced possibility more than once | | | actually constantly | | laid off |
| 25. During the past year, how often were you in a situation where you faced job loss? | 1 | 2 | | 3 | | | 4 | | 5 |
|  | not at all likely | | not too likely | | somewhat likely | | | very  likely | |
| 26. Sometimes people permanently lose  jobs they want to keep. How likely is it  that during the next couple of years you will lose your present job with your employer? | 1 | | 2 | | 3 | | | 4 | |

©Copyright by Robert Karasek JCQ Center. All rights reserved. No distribution without permission from The JCQ Center by Director Robert Karasek


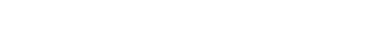

Supplement: Supplementary file 1 — Supplementary Material: Additional file 1 - STROBE Statement—Checklist of items that should be included in reports of cross-sectional studies [file 40359_2024_2279_MOESM1_ESM.docx]
